# European Ethnopharmaceuticals for Self-Medication in Japan: Review Experience of *Vitis vinifera* L., Folium Extract and *Vitex agnus-castus* L., Fructus Extract as OTC Drugs

**DOI:** 10.3390/medicines5010003

**Published:** 2018-01-06

**Authors:** Tatsuro Hoshino, Nanami Muto, Shinsuke Tsukada, Takatoshi Nakamura, Hikoichiro Maegawa

**Affiliations:** 1 Office of Planning and Coordination, Pharmaceuticals and Medical Devices Agency, Tokyo 100-0013, Japan; hoshino-tatsuro@pmda.go.jp; 2 Office of OTC/Quasi-drugs, Pharmaceuticals and Medical Devices Agency, Tokyo 100-0013, Japan; muto-nanami@pmda.go.jp (N.M.); cba07499@nifty.com (T.N.); 3 Office of New drugs IV, Pharmaceuticals and Medical Devices Agency, Tokyo 100-0013, Japan; tsukada-shinsuke@pmda.go.jp; 4 Division of Pharmacognosy, Phytochemistry and Narcotics, National Institute of Health Sciences, Kanagawa 210-9501, Japan; 5 Office of Vaccines and Blood Products, Pharmaceuticals and Medical Devices Agency, Tokyo 100-0013, Japan

**Keywords:** herbal medicine, regulation, OTC, Pharmaceuticals and Medical Devices Agency (PMDA)

## Abstract

Since the publication of “Application Guideline for Western Traditional Herbal Medicines as OTC Drugs” in 2007, only two European ethnopharmaceuticals, *Vitis vinifera* L., folium extract (Antistax) and *Vitex agnus-castus* L., fructus extract (Prefemin), have been approved as OTC drugs in Japan. In this review, we describe the current regulation of Western ethnopharmaceuticals in Japan, summarize our regulatory experiences and discuss the scientific and regulatory issues involved.

## 1. Introduction

The World Health Organization (WHO) strengthens safety, quality and effectiveness of traditional and complementary medicines through regulation, and promotes universal health coverage by integrating traditional and complementary medicine services and self-healthcare into national health systems [1]. In Japan, traditional medicines, of which safety, quality and effectiveness are ensured through regulations, are integrated into the national healthcare system [2,3]. Traditional herbal medicines in Japan are classified into two categories, which are Kampo products and non-Kampo crude drug products. Kampo products are formulated based on Kampo medicine principles, whereas non-Kampo crude drug products contain single or multiple crude drugs, and their formulations do not follow Kampo medicine principles, but folk medicines instead. Kampo products include ethical Kampo formulations and over-the-counter (OTC) Kampo formulations. Ethical Kampo formulations are listed in the National Health Insurance (NHI) price list and are obtained through a doctor’s prescription with NHI reimbursement. Non-Kampo crude drug products can also have ethical status and OTC status. Ethical non-Kampo crude drug products contain single crude drug products and are also listed in NHI Price List. The benefits of OTC drugs include convenience to patients, better self-management of minor illnesses, and a reduction in government medical costs [4]. Therefore, OTC Kampo and non-Kampo crude drug products have a significant role in self-medication.

“Borderline of Pharmaceuticals to Non-Pharmaceuticals” was published by the Ministry of Health and Welfare (MHW; currently Ministry of Health, Labour and Welfare (MHLW)) in 1971. The latest edition of it was published by MHLW in 2016 [5]. Some of the Western herbs have been listed under non-pharmaceuticals, and can be sold as health supplements without review [5,6]. Western herbal products as health supplements do not strictly provide safety information on overdose and quality is unclear and variable. In recent years, public health awareness and self-medication by OTC drugs have grown in Japan. In 2002, the interim report by “The Investigative Committee for Review Rationalization of OTC Drugs” proposed that Western herbal remedies used as medicines should be regulated in Japan [7]. Based on this, “Application Guideline for Western Traditional Herbal Medicines as OTC Drugs” was published in 2007 [8]. Other ethnopharmaceuticals mostly from Europe could be approved as OTC drugs in Japan. The summary of approval criteria for these herbal remedies are below:

∙ They should have OTC drug approval in the country with well-established pharmaceutical regulations.
∙ Their efficacy and safety should be based on scientific evaluation of well-designed clinical trials including some clinical literature.
∙ If the efficacy and safety data of the products have already been assessed in the country with well-established pharmaceutical regulations, this data can be utilized in the approval process in Japan.
∙ Their quality must be comparable with that of drug products used in clinical trials to show their efficacy and safety as indicated above.
∙ Safety to Japanese population is required.
∙ Identification of botanical origin and quality control for crude drugs, crude drug extract and products is required.

Based on the guideline above, Antistax and Prefemin were approved as OTC drugs in January 2011 [9] and in April 2014 [10] respectively. The regulatory information on herbal medicinal products will help to identify what kind of research is needed for evidence-based herbal medicine use [11]. In this review, we summarize our regulatory experiences and discuss the scientific and regulatory issues involved, compared with European Union (EU) guidelines and the United States (US) guidance.

## 2. Antistax

Antistax, containing 360 mg of red vine leaf extract as a daily dose, was approved as an OTC drug in January 2011. It is indicated for swelling of the lower legs, ankles and heavy, achy, tired legs associated with venous disorder caused by long periods of standing or sitting. This product is the same formulation as “Antistax” registered or approved as an OTC drug in Austria and over 20 other countries. Since ancient times, beneficial health effects from vine leaves have been described, and confirmed by numerous “recipes” reported in the writings such as Hippocrates of Cos (5th–4th century BC). Therapeutic literature from France showed the origin of using red leaves from the grape vines “teinturiers”. Grapevine leaves and extract have been traditionally used for the treatment of symptoms associated with venous insufficiency for more than 70 years in France. While the whole extract preparation as such is considered the active agent, it is particularly characterized by its content of flavonol glycosides and glucuronides, i.e., quercetin-3-*O*-β-d-glucuronide, quercetin-3-*O*-β-glucoside, and kaempferol-3-glucoside. These flavonoids are considered to contribute predominantly to the pharmacological effects [12].

### 2.1. Data-Package

The data package for application of Antistax is shown in Table 1. Compared to the data requirements for drugs containing new active ingredients undergoing OTC application process in Japan, tests under severe conditions, secondary pharmacodynamics, safety pharmacology and ADME are omitted.

### 2.2. Quality Control

The crude drug is same as the monograph “Vigne Rouge” listed in French Pharmacopoeia 10th Edition. The red vine leaf dry extract containing additives goes under a quality control (QC) process. The active ingredients red vine leaf extract is combined with light anhydrous silicic acid and dried hydrolyzed starch. The red vine leaf extract is derived from the leaves of red-fleshed European grapes *V. vinifera* of which pericarp is black. The standards of red vine leaves, red vine leaf dry extract and product were in accordance with that of European approved products.

In the review, acid-insoluble ash and extract content of red vine leaves were additionally required for conducting standards and test methods. Loss on drying and total ash were also changed to methods complying with the Japanese Pharmacopoeia (JP). An impurity test (heavy metal, arsenic) and extract content were additionally required for conducting standards and test methods of the red vine leaf dry extract. Also, test methods for loss on drying, total ash and microbial limit were changed in accordance with JP. For the product’s standards and test methods, loss on drying and extract content were required. Test methods for uniformity of dosage units, disintegration and microbial limit were changed in accordance with JP.

A red vine leaf was mainly identified by thin-layer chromatography (TLC) and a red vine leaf dry extract was mainly identified by high-performance liquid chromatography and TLC. In the review, an explanation for not genetically identifying the crude drugs was required. The applicant answered that genetic identification is impossible to use for quality control at that time because there are several varieties of *V. vinifera* species that cannot be distinguished by DNA polymorphism analysis. This applicant’s answer was accepted.

### 2.3. Clinical Trials

Based on “Application Guideline for Western Traditional Herbal Medicines as OTC Drugs”, a placebo-controlled randomized double-blind multicenter clinical trial that has already been assessed abroad for the drug’s approval and an open-label clinical trial for safety in the Japanese population were submitted as a data package (Table 2).

Regarding efficacy, changing the limb volume using water displacement after 12 weeks administration in trial 1 was evaluated as the primary endpoint. In comparison with the placebo group, the limb volume decreased significantly in the treatment group (Table 3).

Regarding safety, in the foreign clinical trial, the side effects observed in this drug group were mild in 2 cases (2.3%, 2/87 cases). In the open-label clinical trial for Japanese, side effects observed were mild in 12 cases (6.7%, 12/180 cases) and were recovering or recovered.

### 2.4. Pharmacological Action

The main tests submitted for primary pharmacodynamics analysis are shown in Table 4. Since pathophysiology of chronic venous insufficiency (CVI) is unclear, red vine leaf extract’s mechanism of action on CVI symptoms has been investigated. The tests submitted showed red vine leaf extract has anti-edematous effects, antihistaminic effects, and effects on the blood coagulation system. The various effects may be attributed to various pharmacology active components in the extract.

## 3. Prefemin

Prefemin containing 20 mg of chest berry extract as a daily dose was approved as an OTC drug in April 2014. It is indicated for mitigation of premenstrual syndrome (PMS): breast swelling, headache, irritation and mood swings. Chest berry was traditionally used by ancient Greeks and Romans for treating various gynecological disorders such as menstruation irregularities and breast pain. This product is the same product as “Prefemin Filmtabletten” approved in Switzerland in 1999 as an OTC drug.

### 3.1. Data-Package

The data package for application of Prefemin is shown in Table 1. Compared to the data requirements for drugs containing new active ingredients undergoing OTC application process in Japan, tests under severe conditions and ADME are omitted.

### 3.2. Quality Control

Chest berry is an herbal medicine listed in several official books (European Pharmacopoeia (EP), Swiss Pharmacopoeia, British Pharmacopoeia, French Pharmacopoeia, German Commission E Monograph and WHO Monograph on selected Medicinal Plants). It is the dry mature fruit of the chest tree (*V. agnus-castus*), and it is listed as “Agnus Castus Fruit” in EP. The chest berry extract is obtained by extraction with ethanol, then granulated and dried. This chest berry dry extract undergoes QC process. Chest berry 60% ethanol dry extract is categorized under “well-established use” in the European Medicines Agency (EMA) monograph.

In the review, the standards and test methods of chest berry, chest berry dry extract and product were required to comply with JP. In addition, explanation of quality equivalence with the product used for the submitted clinical trial were required, because manufacturing method of the chest berry dry extract and the product have changed since the approval in Switzerland.

### 3.3. Clinical Trials

Based on “Application Guideline for Western Traditional Herbal Medicines as OTC Drugs”, two placebo-controlled randomized double-blind multicenter clinical trials that have already been assessed abroad for the drug’s approval and an open-label clinical trial for safety in Japanese population were submitted in a data package (Table 5).

Regarding efficacy, the changes from the baseline to the end of administration of the visual analog scale (VAS) total score of 6 symptoms (irritated feelings, dysthymia, anger, headache, breast pain and abdominal distension) in trial 1 was evaluated as the primary endpoint. Compared with the placebo group, the total VAS score significantly decreased in the treatment groups (Table 6).

Regarding safety, side effects were not observed in foreign clinical trials; there was 1 moderate case of side effects (1.4%, 1/69 cases) in the Japanese clinical trial.

### 3.4 Pharmacological Action

The main tests submitted for primary pharmacodynamics analysis are shown in Table 7. Since the causes of PMS are attributed to hormonal imbalance, hyperprolactinemia seems to be an important factor. Inhibition of prolactin release via interaction with D_2_-subtype dopamine receptor by chest berry extract has been studied. The tests submitted showed chest berry extract mainly affect the dopamine D_2_ receptor.

## 4. Discussion

### 4.1. Efficacy of Herbal Medicines

In the EU, the EMA’s Committee on Herbal Medicinal Products (HMPC) published monographs for herbal medicines. In the monograph, “well-established use” status is given to substances that have been in use in the EU for at least 10 years, and have had recognized efficacy in well-designed controlled clinical study and an acceptable level of safety [13]. The US Food and Drug Administration (FDA) published the Botanical Drug Development Guidance for Industry [14]. In this guidance, Phase 3 clinical studies of botanical drugs have the same purpose as Phase 3 clinical studies of non-botanical drugs. Specific to botanical drugs, analyses of batch effects on clinical endpoints should be considered when drug product batches exhibit variations, potentially affecting clinical outcomes. In the first US-approved botanical drug Veregen, two randomized, placebo-controlled, double-blind clinical trials demonstrate scientifically consistent efficacy with different batches [15]. Making changes to manufacturing methods during clinical development could change the chemical profile of the drug substance in the resulting botanical drug product and may warrant bridging studies to justify reliance of previous clinical testing results [14]. Therefore, it is necessary to demonstrate efficacy in randomized controlled trials using application products or products having quality equivalence with application products to approve herbal products as medicines.

### 4.2. Data-Package of Herbal Medicines

Omission of some parts of the application data can be scientifically acceptable due to the characteristics of the herbal medicinal products in “Application Guideline for Western Traditional Herbal Medicines as OTC Drugs” [8]. Data for ADME are not required for both Antistax and Prefemin approvals [9,10]. It is unnecessary to submit ADME data mainly because botanical drugs that are multicomponent systems are not expected to show efficacy by specific chemical compounds, or identifiable active ingredients. There is no data on pharmacokinetics in the EMA assessment reports on *V. vinifera* and *V. agnus-castus*, as “well-established use” [12,16]. Also, the FDA guidance states it is likely that more than one chemical constituent in a botanical drug or the active constituents may not be identified, standard *in vivo* bioavailability and pharmacokinetic studies that measure the blood or urine concentration of the active moieties or active metabolites may be difficult or impossible to perform [14]. Therefore, omission of pharmacokinetics data can be acceptable. However, explanations of drug interactions were required in the review of Antistax and Prefemin [9,10]. It is well-known that herbal remedies such as St John’s wort have clinically significant interactions with prescribed medicines including warfarin, cyclosporin, theophylline resulting in a decrease in the concentrations or effects of the medicines through the induction of cytochrome P450 isoenzymes such as CYP3A4 and CYP1A2 [17]. Drug interactions of herbal medicines should be examined and collecting drug interaction information in post marketing surveillance is important.

### 4.3. Quality Control of Herbal Medicines

In the EU, to ensure the quality of crude drugs, botanical raw materials are processed in accordance with the guideline on good agricultural and collection practice (GACP) for starting materials of herbal origin [18]. In the FDA guidance, botanical raw materials are also processed in accordance with GACP [14]. Also, botanical raw material from representative cultivation sites or farms are important for the manufacturing of the clinical drug substance for multiple batch Phase 3 studies. Starting materials of Veregen and Fulyzaq must be collected in specified sites [19,20]. Self-imposed GACP is published by the Japan Kampo Medicines Manufacturers Association (JKMA) in Japan [21]. The first step in the quality assurance of natural products is the use of raw materials from the right origin (and the right source). Therefore, it is clearly stated in JP17 Article 4 of the General Rules for Crude Drugs that the origin of crude drugs is to serve as the acceptance criteria [22]. In the review of Antistax, identification of the botanical raw material origin and an explanation of the reason for not genetically identifying crude drugs were required [9]. Together with recent progress in molecular biology techniques and the accumulation of genetic information on plants, differentiating methods of crude drugs based on genotypes have been established. Unlike morphological and other methods that are based on phenotypic characteristics, the genotypic methods are not affected by environmental factors. Also, the methods have several advantages; specialized expertise and skill for classification are not needed, and objective results are easily obtained. Purity Tests on Crude Drugs Using Genetic Information has been listed as General Information since JP15 Edition Supplement I. In the EMA Guideline on quality of herbal medicinal products/traditional herbal medicinal products [23], genetic classification is not mentioned on the identification of raw material origin. However, the FDA guidance encourages the development of a genetic taxonomic method [14]. In the case of Veregen, genetic information was used as one of the identification criteria for botanical raw material origin [19]. From the viewpoint of quality assurance, genetic information will be used more for identification of botanical raw material origin in the future.

Because herbal extracts are multicomponent systems and whole herbal extracts are regarded as active ingredients, quality control for whole herbal extracts is required. Regarding standards and test methods of herbal extracts, “Application Guidance for OTC non-Kampo Crude Drug Extract Products in Japan [24]” is compared with “Guidelines on specifications of EMA: guidelines on specifications: test procedures and acceptance criteria for herbal substances, herbal preparations [25]” and the FDA guidance [14] (Table 8).

The criteria to be set in standards and test methods of herbal extracts are generally similar in Japan, the US and the EU. However, to manage the whole herbal extract, mass balance and biological assay in US, and extract content in Japan are specific to the respective countries. In the review report of Veregen, biological assays were discussed as follows: bioassays can be used in combination with established chemistry, manufacturing and control (CMC) specifications to compare the similarity of botanical raw materials when adding new cultivars, changing providers of previously used cultivars, or implementing other manufacturing changes. A more comprehensive approach, such as combining bioactivity equivalence and CMC specifications, will be preferable to only the CMC specifications. The most desirable bioassays would include those that are able to correlate the bioactivity of the drug substance with the clinical effects [19]. Also, in the review of Fulyzaq, the possibility of developing an *in vitro* pharmacological test as a bioassay for quality control was discussed. Finally, evaluation of the possibility of establishing a clinically relevant bioassay for qualifying future manufacturing changes are required in post-approval [26]. In the future, if there is any feasibility *in vitro* bioassays that can correlate biological activity of herbal extract with clinical effect, bioassay as standards and test methods of herbal extract may be one of the options for quality control to evaluate quality equivalence when changing the manufacturing method or to ensure quality consistency.

### 4.4. Issues in Herbal Medicines’ Development

In the ten years have passed since the publication of “Application Guideline for Western Traditional Herbal Medicines as OTC Drugs”, only two products have been approved as OTC drugs in Japan. One of the reasons may be that applicants do not understand the appropriate data packages for the application. In the review of Western herbal medicines, the main issues were appropriate indications of foreign application products as OTC drugs in Japan and the regulatory status in foreign countries. As discussed above, there are many issues with efficacy and quality equivalence. These issues include manipulation of clinical trial data and manufacture changing application products post clinical trial. For efficient development of Western herbal medicines, applicants should consider these issues before approaching the regulatory agency.

## 5. Conclusions

In Japan, since the publication of “Application Guideline for Western Traditional Herbal Medicines as OTC Drugs” in 2007, two European ethnopharmaceuticals, *V. vinifera* extract and *V. agnus-castus* extract, have been approved as OTC drugs in Japan. In this review, our regulatory experiences are summarized. It is difficult to describe all regulatory issues because we would like to focus on specific herbal medicine issues. Please refer to the review reports for in-depth details of the review, although please note that the reports are written in Japanese. Comparing Japanese regulations with the regulations of the EU and the US, the concept of regulation of herbal medicines seems similar among Japan, the EU and the US. For improvements in public health, we hope that the regulatory information about traditional herbal products in Japan will contribute towards tackling the challenging task of regulating traditional herbal products worldwide.

## Figures and Tables

**Table 1:** Data-package of Antistax and Prefemin in Japan, and comparison of the data requirements for Western traditional herbal medicines as OTC drugs.

Contents of the Data Submitted for Application		Antistax	Prefemin	Western Traditional Herbal Medicines as an OTC Drug ^(1)^
A. Origin or background of discovery, conditions of use in foreign countries	Origin or background of discovery	○	○	○
	Conditions of use in foreign countries	○	○	○
	Therapeutic group, comparisons with other drugs, and related information.	○	○	○
B. Manufacturing methods, standards and test methods	Chemical structure, physicochemical properties, and related information.	○	○	○
	Manufacturing methods	○	○	○
	Standards and test methods	○	○	○
C. Stability	Long-term storage tests	○	○	○
	Tests under severe conditions	×	×	○
	Accelerated tests	○	○	○
D. Pharmacological action	Primary pharmacodynamics	○	○	○
	Secondary pharmacodynamics, Safety pharmacology	×	○	○
	Other pharmacological action	×	○	△
E. Absorption, distribution, metabolism, and excretion	Absorption	×	×	○
	Distribution	×	×	○
	Metabolism	×	×	○
	Excretion	×	×	○
	Bioequivalency	×	×	×
	Other ADME	×	×	△
F. Acute, subacute, and chronic toxicity, teratogenicity, and other type of toxicity	Single dose toxicity	○	○	○
	Repeated dose toxicity	○	○	○
	Genotoxicity	○	○	○
	Carcinogenicity	×	×	△
	Reproductive toxicity	○	○	○
	Local irritation	×	×	△
	Other toxicity	×	×	△
G. Clinical studies	Clinical trial results	○	○	○
H. Package insert	Package insert	○	○	○

In principle, ○ means that the indicated data is required. × means that the indicated data is not required. △ indicate necessity of the indicated data depending on characteristics of applicant products. ^(1)^ In the case of products with new active ingredients.

**Table 2:** Summary of clinical trials.

No.	Region	Study Design	Population	Number of Subjects	Dose (mg/Day) as Red Vine Leaf Extract	Summary of Dosage and Administration Method
1	Foreign	Multicenter randomized Placebo-controlled double-blind	Patients with chronic venous insufficiency (Widmer class I and II) (aged 25 to 75 years)	260	Placebo	Once a day, 12 weeks
					360	
					760	
2	Japan	Open-label	Patients have various symptoms due to reflux vein disorder * (aged 20 years and older)	180	360	Once a day, 12 weeks

* Symptoms of “weight feeling/fatigue (dullness), tightness, tingling feeling, pain, hot feeling, itching”.

**Table 3:** Efficacy of Antistax in the trial 1.

Group (Number of Subjects)	Changing Limb Volume Using Water Displacement in 12 Weeks after Administration
Placebo group (*n* = 87)	+33.7 ± 96.1 g
360 mg/day group (*n* = 86)	−42.2 ± 74.6 g
720 mg/day group (*n* = 84)	−66.2 ± 108.9 g

**Table 4:** Summary of primary pharmacodynamics tests.

Effect	Test System	Group	Test Method	Results
Anti-edematous effect	*In vivo* (rat)	Red vine leaf extract ^(1)^ 100 mg/kg, 1000 mg/kg, water (control group)	The effect on histamine-induced paw edema was examined.	Edema was suppressed in both extract treatment groups.
	*In vivo* (rat)	Red vine leaf extract ^(^^1)^ 10 mg/kg, 100 mg/kg, water (control group)	The effect on carrageenan-induced paw edema was examined.	Edema was significantly suppressed in the 100 mg/kg group.
	*In vivo* (rat)	Red vine leaf extract ^(1)^ 10 mg/kg, 100 mg/kg, water (control group)	The effect on egg white edema of rats paw was examined.	Edema was slightly suppressed in the 100 mg/kg group.
Antihistaminic effect	*In vitro* (ileum isolated from female guinea pig)	Red vine leaf extract ^(1)^ 10^−4^, 3 × 10^−4^, 10^−3^ g/mL	The effect of histamine on smooth muscle contraction was examined.	The contraction of the ileum was suppressed in a dose-dependent manner, and a significant antihistaminic effect was observed at 3 × 10^−4^ g/mL or more.
Effect on the blood coagulation system	*In vivo* (rat)	Red vine leaf extract ^(1)^ 10, 100 mg/kg, water (control group)	The effect on bleeding time was examined.	Bleeding time was significantly prolonged at 100 mg/kg compared with the control group.
	*In vitro* (rat platelet rich plasma)	Red vine leaf extract ^(2)^ 6, 20, 60 µL/mL	The effect on arachidonic acid-induced platelet aggregation was examined.	Platelet aggregation was inhibited in dose-dependent manner, and a significant inhibitory effect was observed at 60 µL/mL.

^(1)^ Concentrated red vine leaf extract (Residue after drying, which is about 60% of extract); ^(2)^ Extracts from 5 g of red vine leaves per 200 mL of water.

**Table 5:** Summary of clinical trials.

No.	Region	Study Design	Population	Number of Subjects	Dose (mg/Day) as Chest Berry Extract	Summary of Dosage and Administration Method
1	Foreign	Multicenter randomized Placebo-controlled double-blind	Patients with PMS (aged 18 to 44 years)	178	Placebo	Once a day 3 menstruation cycles
					20	
2		Multicenter randomized Placebo-controlled double-blind (dose response study)	Patients with PMS (aged 18 years and older)	162	Placebo	Once a day 3 menstruation cycles
					8	
					20	
					30	
3	Japan	Open-label	Patients with PMS (aged 18 to 44 years)	69	20	Once a day 3 menstruation cycles

**Table 6:** Efficacy of Prefemin in the trial 1.

Primary Endpoint	Average of Baseline		Average of Changes from Baseline		
	20 mg/Day (*n* = 86)	Placebo (*n* = 84)	20 mg/Day (*n* = 86)	Placebo (*n* = 84)	*p* Value
6 Symptom total VAS score (mm)	263	256	−128.5	−78.1	0.001

**Table 7:** Summary of primary pharmacodynamics tests.

Effect	Test System	Group	Test Method	Results
Dopaminergic receptor binding activity (D_2_ receptor)	*In vitro* (calf striatal sample)	Chest berry extract 5 lots	[^3^H]–spiperone ^(1)^ binding inhibition test	The IC_50_ (µg/mL) of 5 lots was almost equivalent (40 to 69), and each extract lot was shown to have dopaminergic receptor binding activity (D_2_ receptor).
Stimulatory effects of dopamine D_2_ receptor	*In vitro* (rat striatal specimen incorporating [^3^H]-choline)	Chest berry extract group (70 µg/mL), chest berry extract (70 µg/mL) + spiperone (3 × 10^−9^, 3 × 10^−8^ or 3 × 10^−7^ mol/L) group, control group	Examination of stimulatory effect on dopamine D_2_ receptor with suppression of [^3^H]-choline release by electrical stimulation	Chest berry extract inhibited the release of [^3^H]-choline and this effect was inhibited by spiperone, so this extract showed stimulatory effects of dopamine D_2_ receptor.
Other pharmacological effect via the dopamine receptor	*In vitro* (CHO cell expressing dopamine D_3_ receptor)	Chest berry extract (0.001–10 µg/mL)	7-hydroxy [^3^H]–DPAT ^(2)^ binding inhibition test	Chest berry extract was shown to have dopaminergic receptor binding activity (D_3_ receptor).
Effects on electroencephalogram and momentum in rat	*In vivo* (rat)	Chest berry extract (10, 25, 50 mg/kg)	Chest berry extract was orally administered to examine the brain waves and momentum of the frontal cortex and the striatum	Brain waves changed with chest berry extract, and momentum was increased (suppressed momentum decrease with time). Dopamine D_2_ receptor antagonists inhibited changes in brain waves in the frontal cortex, but changes in brain waves and momentum in the striatum were uninhibited. These results suggest that the effect in the frontal cortex were mediated through the dopamine D_2_ receptor. However, the effect in the striatum were mediated through receptors other than dopamine D_2_ receptor.

^(1)^ Dopamine D_2_ receptor antagonist; ^(2)^ Dopamine D_3_ receptor ligand.

**Table 8:** Comparison of criteria for standards and test methods of herbal extracts in Japanese Guidance with EU guideline and US guidance.

Criteria	Japanese Guidance[24]	EMA Guideline [25]	FDA Guidance [14]
Properties	○ (Appearance, smell, taste)	○ (containing organoleptic characters)	○ (Appearance)
Identification test	○	○	○
Moisture content	×	○	×
Assay (content)	○	○	○
Impurity test	○ (heavy metal, arsenic, residual pesticide)	○ (residual solvents, heavy metals, microbial limit, mycotoxins, pesticides)	○ (residual pesticides, elemental impurities, residual solvents, radioisotope, microbial limit, adventitious toxins)
Loss on drying	○	×	○
Biological assay	×	×	○
Mass balance ^(1)^	×	×	○
Total Ash	○	×	×
Acid-insoluble ash	○	×	×
Extract content	○	×	×

In principle, ○ means required criteria, or criteria to be set. × means unnecessary criteria or not mentioned. ^(1)^ Quantifying other class of compounds (e.g., lipid, protein) that contribute to the mass balance of the botanical substance.
